# Stromal Expression of the Core Clock Gene *Period 2* Is Essential for Tumor Initiation and Metastatic Colonization

**DOI:** 10.3389/fcell.2020.587697

**Published:** 2020-10-02

**Authors:** Lee Shaashua, Shimrit Mayer, Chen Lior, Hagar Lavon, Alexander Novoselsky, Ruth Scherz-Shouval

**Affiliations:** Department of Biomolecular Sciences, Weizmann Institute of Science, Rehovot, Israel

**Keywords:** circadian clock, *Per2*, tumor microenvironment, colon cancer, chronobiology, metastasis, liver, MC38 colon cancer cells

## Abstract

The circadian clock regulates diverse physiological processes by maintaining a 24-h gene expression pattern. Genetic and environmental cues that disrupt normal clock rhythms can lead to cancer, yet the extent to which this effect is controlled by the cancer cells versus non-malignant cells in the tumor microenvironment (TME) is not clear. Here we set out to address this question, by selective manipulation of circadian clock genes in the TME. In two different mouse models of cancer we find that expression of the core clock gene *Per2* in the TME is crucial for tumor initiation and metastatic colonization, whereas another core gene, *Per1*, is dispensable. We further show that loss of *Per2* in the TME leads to significant transcriptional changes in response to cancer cell introduction. These changes may contribute to a tumor-suppressive microenvironment. Thus, our work unravels an unexpected protumorigenic role for the core clock gene *Per2* in the TME, with potential implications for therapeutic dosing strategies and treatment regimens.

## Introduction

The circadian clock is an endogenous, evolutionally conserved and ubiquitously expressed pacemaker, consisting of cell autonomous clocks and a central pacemaker located in the hypothalamus suprachiasmatic nucleus (SCN). Together these clocks synchronize numerous biological processes between the organism and its environment. Amongst these processes are DNA damage repair, metabolism, and cell cycle (Blakeman et al., 2016). The circadian clocks act as oscillators to drive 24-h rhythms in gene expression and protein function, and these rhythms help the organism maintain a homeostatic relationship with the environment (Padmanabhan and Billaud, 2017). Disruption of circadian rhythms has been associated with various forms of cancer in humans, and it has been shown that a disordered circadian clock, whether genetically or due to environmental signals (e.g., changes of dark/light exposure) accelerates tumor progression (Savvidis and Koutsilieris, 2012; Hadadi et al., 2019). Additionally, epidemiological studies show that women who work in irregular shift work may be at higher risk of developing breast cancer (Schernhammer et al., 2003; Hansen and Stevens, 2012; Blakeman et al., 2016). Moreover, the toxicity and efficacy of various cancer therapies is dictated by time-of-day (Kobayashi et al., 2002; Lauriola et al., 2014; Borniger et al., 2017). Cancer cells with a disrupted clock show increased growth in culture, and mice with a disrupted clock tend to develop more radiation-induced tumors than wild type (WT) mice (Shostak, 2017). Mutations in genes that regulate the molecular clock have been found in human cancer samples, indicating a gene-specific causal relation between the circadian clock and cancer (Kettner et al., 2014). At the cellular level, most cancer cell-lines show loss of rhythmicity, specifically of genes that are related to proliferation and apoptosis, thus enabling their uncontrolled proliferation (Relógio et al., 2014).

The currently held molecular model for circadian rhythmicity is based on a transcription-translation feedback loop. This mechanism includes the transcription factors BMAL1 and CLOCK that drive transcription of the Period (*Per*) and Cryptochrome (*Cry*) genes. Then, PER and CRY proteins dimerize, translocate to the nucleus and repress *Clock-Bmal1* transcription. This orchestrated machinery results in periodic expression of approximately half of the mammalian genes (Zhang et al., 2014). Previous studies found that *Per1* and *Per2* are essential for the proper function of the circadian clock, while the contribution of *Per3* is not as important (Bae et al., 2001). *Per2* was shown to be essential for proper function of the circadian clock under conditions of constant dark or light, as mice lacking *Per2* exhibit a faulty clock that is reflected by a shorter period and rhythmic instability (Zheng et al., 1999; Tamiya et al., 2016). *In vitro*, loss of *Per1* or *Per2* was suggested to have a much milder effect on cell rhythmicity (Ramanathan et al., 2014), compared to the effect observed *in vivo*.

It is well established that the non-malignant components of the tumor, also termed the tumor microenvironment (TME) are an integral and essential part of the tumor. The TME is composed of many cell types, including immune cells, cancer associated fibroblasts (CAF), endothelial cells, and extracellular matrix (ECM). The TME is essential for tumor formation, homeostasis, and progression (Quail and Joyce, 2013; Yuan et al., 2016; Jin et al., 2017). It is dynamically reshaped, thus certain cell populations in the TME can be detrimental for early stages of tumor initiation, while protumorigenic in later stages of tumor progression (Lu et al., 2012; Bercovici et al., 2019; Lin et al., 2019; Wei et al., 2020). Previous studies have shown that cancer cells within a tumor lose their rhythmicity, yet there is sparse data about rhythmicity of the TME. This is partially due to the fact that the vast majority of published studies on cancer and circadian clocks used mouse models in which both the TME and the cancer cells are affected by a disrupted clock, either by genetic mutations or by environmental cues. Recently it was shown that mice with environmentally disrupted clocks had a more protumorigenic immune microenvironment in breast cancer (Hadadi et al., 2019) and thoracic cancer (Yang et al., 2019). Pan-cancer analysis supported these findings, and suggested that circadian genes are involved in the regulation of cancer immunity (Zhou et al., 2020). Not only the immune microenvironment, but also CAFs were proposed to play a role in clock regulation in cancer, when shown that co-culturing colon cancer cells with CAFs improved the rhythmicity of cancer cells (Fuhr et al., 2019). Gaining a better understanding of how circadian clocks in the TME affect the development and progression of the tumor could lead to novel therapeutic advances and approaches. It is possible that synchronization, or lack of synchronization, between the TME and the cancer cells, plays a role in cancer progression. For example, tumors may rely on the TME maintaining intact circadian rhythms to compensate for imbalances resulting from loss of rhythmicity in the cancer cells. Alternatively, loss of rhythmicity may be essential for cells of the TME to shift from antitumorigenic to protumorigenic. In this study we set out to explore the role of the circadian clock in the TME. Using orthotopic injection of cancer cells into clock-deficient mice, we find that stromal *Per2*, but not *Per1*, is required for tumor growth and metastatic colonization. Loss of *Per2* in the TME leads to transcriptional rewiring at early stages of metastases formation, and suppresses subsequent metastatic tumor progression, highlighting *Per2* as an unexpected protumorigenic mediator in the TME.

## Results

### The Circadian Rhythm in Cancer Cells Is Disrupted, While Normal Fibroblasts Maintain a Robust Rhythm, *in vitro*

**FIGURE 1:**
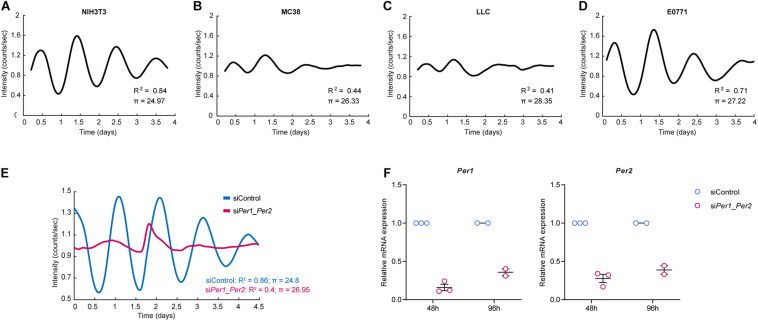
Cancer cells show disrupted rhythmicity compared to normal fibroblasts. **(A–D)** Per2::luc expressing NIH3T3 fibroblasts or cancer cells, as indicated, were synchronized by dexamethasone and their luminescence signal was measured continuously (every 12 min) in a LumiCycle luminometer. The period and non-linear regression model coefficient of determination are presented (see “Materials and Methods”). The curves are an average of 3 technical repeats, and are representative of at least two independent experiments. **(E,F)** *Per1* and *Per2* were knocked down in Per2::luc-expressing NIH3T3 cells using siRNA. The cells were synchronized by dexamethasone and their luminescence was measured continuously (every 12 min) in a LumiCycle luminometer. **(E)** Period and the non-linear regression model coefficient of determination are presented. The curves are an average of 3 technical repeats, and are representative of two experiments. **(F)** Validation of *Per* gene knockdown efficiency at two time points by RT-PCR is presented as mean ± SEM.

A disrupted circadian clock is associated with tumorigenesis, and cancer cells are thought to express a less robust clock compared to non-malignant cells (Welsh et al., 2004; Kiessling et al., 2017). To test this, we set out to evaluate the rhythmicity of NIH3T3, a non-malignant immortalized fibroblast cell line, and of three cancer cell lines – MC38 (colon), LLC (lung), and E0771 (breast) – using continuous bioluminescence monitoring of cells. Cells were transduced with a *Per2* promotor-driven luciferase reporter (Yoo et al., 2004; Liu et al., 2008), synchronized by the synthetic glucocorticoid Dexamethasone, and longitudinally assessed using a LumiCycle luminometer (Yamazaki and Takahashi, 2005). To better understand the rhythmic behavior of cells, we developed a regression model that calculates the period length (π) of the curve (see “Materials and Methods”). The coefficient of determination *R*^2^ can be used as a readout for how closely our empiric data follows a sinusoidal pattern. As evident by the period and the coefficient of determination, NIH3T3 fibroblasts exhibited robust rhythmicity (Figure 1A), while all three cancer cell lines were less rhythmic than NIH3T3 (Figures 1B–D). E0771 cells exhibited the highest coefficient of determination of all three cancer cell lines, indicating that their rhythmicity is the most intact, while still less robust than that of non-malignant cells. The period length of all three cancer cell lines was longer than that of the fibroblasts, with LLC having the longest period (28.3 h), followed by E0771 (27.2 h), and MC38 (26.3 h). Next, we asked whether the rhythmicity of the NIH3T3 fibroblasts could be disrupted by loss of *Per1/Per2*. We knocked down *Per1* and *Per2* using siRNA and assessed their rhythmicity using bioluminescence. Indeed, we found that deletion of *Per1* and *Per2* impairs cell rhythmicity (Figures 1E,F). These results suggest that the rhythmicity of cancer cells is disrupted, *in vitro*, while normal cells maintain a functional clock, the activity of which is dependent on the expression of the core clock genes *Per1* and *Per2.*

### Loss of *Per1* and *Per2* Inhibits Metastatic Colonization

**FIGURE 2:**
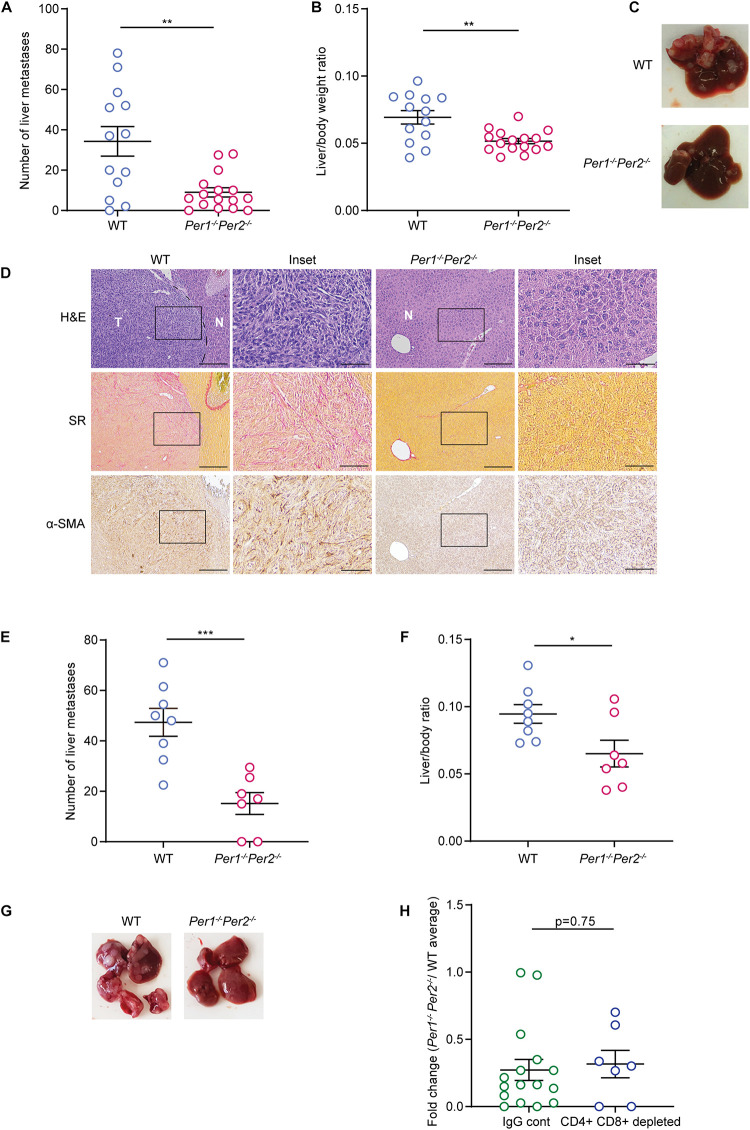
Clock deficient mice exhibit lower metastatic burden. **(A–D)** WT and *Per1*^–/^*^–^Per2^–^*^/^*^–^* mice were injected with MC38 cancer cells into the hepatic portal vein. At 21 days post injection macrometastases were quantified by visual inspection **(A)** and the ratio of liver weight to body weight was calculated **(B)**. *n* = 13–16 mice per genotype combined from three independent experiments. *P*-value was calculated using unpaired Student’s *t*-test with Welch’s correction. Error bars represent SEM. **(C)** Representative images of livers from WT and *Per1*^–/^*^–^Per2^–^*^/^*^–^*mice. **(D)** Representative Hematoxylin and Eosin (H&E), Sirius red (SR), and α-SMA staining of livers from WT and *Per1*^–/^*^–^Per2^–^*^/^*^–^* mice. Scale bar – 200 μm; inset – 67 μm; T-tumor, N-normal tissue. **(E–H)** WT and *Per1*^–/^*^–^Per2^–^*^/^*^–^* mice were injected with antibodies of CD4+ and CD8+ 2 days prior to MC38 injection and every 5 days until the end point. At 21 days post injection macrometastases were quantified by visual inspection **(E)** and the ratio of liver weight to body weight was calculated **(F)**. *n* = 7–8 per genotype combined from two independent experiments. *P*-value was calculated using unpaired Student’s *t*-test. **(G)** Representative images of livers from WT and *Per1*^–/^*^–^Per2^–^*^/^*^–^* mice with T cell depletion are shown. **(H)** The number of metastases in normal and T cell depleted *Per1*^–/^*^–^Per2^–^*^/^*^–^* mice was normalized to the average number of metastases in WT mice. *P*-value was calculated using unpaired Student’s *t*-test. In all figures error bars represent SEM, *p < 0.05, **p < 0.005, ***p < 0.0005.

Several reports have shown that depletion of the *Per* family genes enhances cancer cell growth and tumor progression (Fu et al., 2002; Gery et al., 2006; Yang et al., 2009; Papagiannakopoulos et al., 2016). To understand how loss of rhythmicity in the TME affects tumor growth, we used a mouse model of liver metastasis, employing a syngeneic colorectal cancer cell line (MC38) in C57Bl/6 mice. Since the liver functions in a circadian manner (Reinke and Asher, 2016, 2019), and is affected directly by the feeding patterns of the organism, it serves as a good model to investigate the circadian clock *in vivo* (Tahara and Shibata, 2016). In our model, 20,000 cancer cells were injected to the hepatic portal vein of mice, producing liver metastases 21 days post injection. We applied this model to WT and *Per1^–/–^Per2^–/–^* mice to assess the effect of a clock-impaired TME on metastatic colonization. Surprisingly, *Per1^–/–^Per2^–/–^* mice had a lower metastatic burden than WT mice, as measured by the reduced number of metastases and lower liver/body weight ratio (Figures 2A–C and Supplementary Figure 1A). WT mice developed dozens of liver metastases, which exhibited substantial ECM rearrangements, as evident by Sirius red staining for collagen, and were heavily infiltrated by α-SMA-positive CAFs (Figure 2D). In stark contrast, the livers of *Per1^–/–^Per2^–/–^* mice had significantly fewer metastases, and stained mostly negative for α-SMA and Sirius Red (Figure 2D). These results suggest that loss of *Per* genes in the stroma inhibits metastatic colonization and ECM-remodeling of the metastatic niche.

T-cells are a major component of the TME, and have a key role in the cytotoxic activity against cancer cells. To assess whether T cells mediate the tumor growth inhibition observed in *Per1^–/–^Per2^–/–^* mice, as well as to exclude the possibility that *Per1^–/–^Per2^–/–^* mice reject the MC38 cancer cells due to their expression of *Per1* and *Per2* genes, we injected cancer cells into WT or *Per1^–/–^Per2^–/–^* mice in which T cells were depleted by CD4 and CD8 antibodies (Supplementary Figure 1B). CD4 and CD8 antibodies were injected 2 days prior to the injection of cancer cells, and every 5 days until the endpoint, and the efficiency of depletion was verified by FACS analysis (Supplementary Figure 1B). *Per1^–/–^Per2^–/–^* mice were significantly more resistant to metastatic colonization than WT mice, even upon T cell depletion (Figures 2E–G). The effect of *Per1^–/–^Per2^–/–^* loss in the TME was similar in the presence and absence of T cells (Figure 2H). Together, these results suggest that T cells do not mediate stromal *Per1^–/–^Per2^–/–^*-associated tumor inhibition.

### Stromal *Per2* Regulates Tumor Progression

**FIGURE 3:**
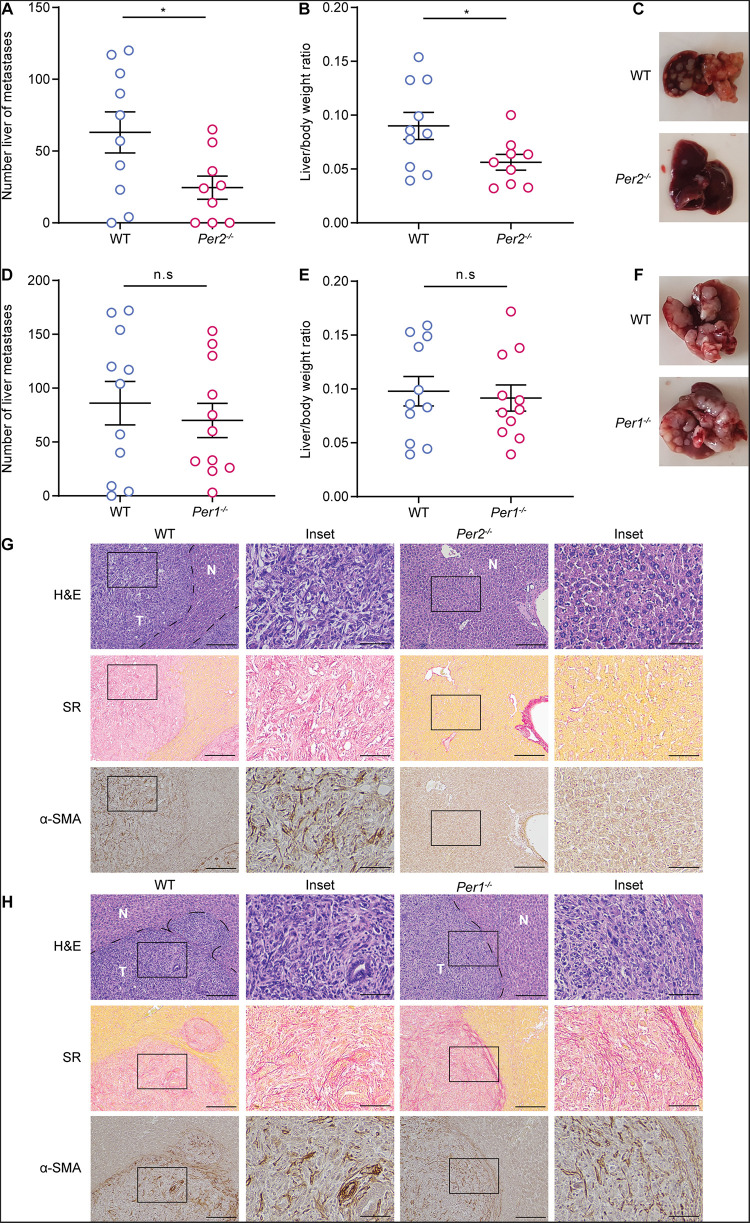
Stromal *Per2*, and not *Per1* promotes metastatic colonization. **(A–C)** WT and *Per2*^–/^*^–^* mice were injected with MC38 cancer cells into the hepatic portal vein. At 21 days post injection **(A)** macrometastases were quantified by visual inspection and **(B)** the ratio of liver weight to body weight was calculated. *n* = 9–10 mice per genotype combined from three independent experiments. **(C)** Representative images of livers from WT and *Per2*^–/^*^–^* mice are shown. **(D–F)** WT and *Per1*^–/^*^–^* mice were injected with MC38 cancer cells into the hepatic portal vein. At 21 days post injection macrometastases were quantified by visual inspection **(D)** and the ratio of liver weight to body weight was calculated (**E**). *n* = 11 mice per genotype combined from three independent experiments. **(F)** Representative images of livers from WT and *Per1*^–/^*^–^* mice are shown. **(G,H)** Representative H&E, Sirius red (SR), and α-SMA staining of livers from WT, *Per2*^–/^*^–^* and *Per1*^–/^*^–^* mice harvested at day 21 post injection. Scale bar – 200 μm, inset – 67 μm; T-tumor N-normal tissue. *P*-value was calculated using unpaired Student’s *t*-test. Error bars represent SEM, *p < 0.05.

It is well established that both *Per1* and *Per2* are essential for maintaining proper circadian clock activity (Bae et al., 2001). To test whether both genes are also essential for the stromal regulation of tumor progression, we injected MC38 cancer cells to the portal vein of mice in which each of the *Per* genes (*Per1, Per2*) was knocked-out separately, and assessed metastatic colonization. WT mice were used as control. *Per2*^–/^*^–^* mice exhibited significantly lower metastatic burden than WT mice (Figures 3A–C). Loss of *Per1*, on the other hand, did not affect metastasis, and *Per1*^–/^*^–^* mice exhibited similar metastatic burden to that of WT (Figures 3D–F). *Per2*^–/^*^–^* livers also showed less collagen deposition and CAF infiltration than WT (Figure 3G), while the amount and the structure of collagen and infiltrating CAFs was similar between *Per1*^–/^*^–^* and WT livers (Figure 3H). These results suggest that stromal *Per2* is essential for metastatic colonization, while *Per1* is dispensable, thus implying that the effect of stromal *Per2* knock-out on metastatic colonization may be clock-independent.

### Transcriptional Analysis of WT and *Per2*^–/–^ Livers Suggests a Role for *Per2* in Eliciting a Pre-metastatic Niche

**FIGURE 4:**
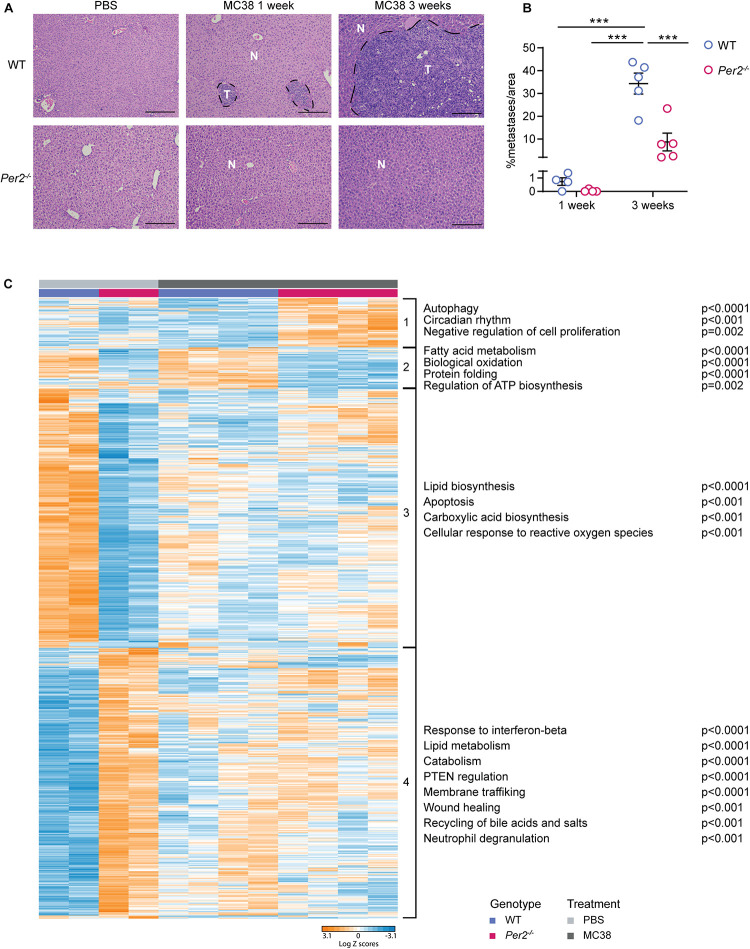
Stromal *Per2*^–/–^ is essential for the formation of the pre-metastatic niche. WT and *Per2*^–/^*^–^* mice were injected with MC38 cancer cells into the hepatic portal vein and sacrificed 1 or 3 weeks post injection, as indicated. PBS was injected to control mice which were sacrificed 1 week post injection. *n* = 4–5 mice per genotype. **(A)** Representative H&E staining of livers from WT and *Per2*^–/^*^–^* mice post injection of PBS or MC38 cancer cells. Scale bar – 200 μm, inset – 67 μm; T-tumor, N-normal tissue. **(B)** The area covered by metastases in MC38-injected livers was calculated by QuPath based on H&E images. *P*-value was calculated using two way ANOVA. Error bars represent SEM, ***p < 0.0005. **(C)** RNA-sequencing was performed on livers of WT and *Per2*^–/^*^–^* mice injected with MC38 (*n* = 4 per genotype) and PBS (*n* = 2 per genotype). Heatmap showing hierarchical clustering of 1222 differentially expressed genes between all four conditions. Pathway analysis was performed using Metascape. Significant pathways are shown; *p* < 0.05, FDR < 0.05 (for details see Supplementary Table 2).

We hypothesized that stromal *Per2* plays a role in preparing the hepatic niche for the initiation of metastasis. To test this hypothesis, we injected MC38 cancer cells (or PBS, as control) to the portal vein of *Per2*^–/–^ and WT mice, harvested the livers 1 week following injection, and performed RNA-sequencing on total liver extracts (Supplementary Table 1). At this early stage only micrometastases (∼0.0005 mm^3^), and not macrometastases (∼5 mm^3^ at 3 weeks) were visible in WT livers (Figure 4A). The average area covered by metastases in the liver was 0.7% in WT mice and 0.05% in *Per2*^–/–^ mice at this early time point (compared to 34.3% in WT mice and 8.7% in *Per2*^–/–^ mice at 3 weeks post injection; Figure 4B). Therefore, we assume that the sequencing data represents mostly the normal liver and the pre-metastatic niche, and not cancer cells. Principal component analysis (PCA) showed that the basal transcriptional landscape of WT and *Per2*^–/–^ livers is different, and further changes following injection of cancer cells (Supplementary Figure 2B). Hierarchical clustering highlighted 1222 differentially expressed genes between WT and *Per2*^–/–^ livers injected with PBS or with MC38 cancer cells (Figure 4C and Supplementary Table 1). In the control mice (PBS), loss of *Per2*^–/–^ led to upregulation of the response to interferon-beta (*Ifit1, Stat1, Tgtp1, Eif2ak2*), catabolism (*Adam9, Pik3c2a, Tnfaip3, Rock1*), wound healing (*Cd36, Cldn1, Pdpn, Pdgfra, Pten*), recycling of bile acids and salts (*Slc10a2, Abcb11, Slco1b2*), and neutrophil degranulation (*Adam10, Ctsc, Degs1*; Figure 4C and cluster 4 and Supplementary Table 2). Apoptosis (*Akt1, Bad, Bcl2l1, Bnip3*), carboxylic acid biosynthesis (*Aldoa, Apoa1, Apoa4, Ccnd3*), and the cellular response to reactive oxygen species (*Sod3, Pdk2, Park7, Ccs*) were downregulated in the *Per2*^–/^*^–^* livers (Figure 4C and cluster 3 and Supplementary Table 2). The same genes and pathways were also downregulated (though to a lesser extent) following injection of MC38 cells to either WT or *Per2*^–/^*^–^* mice, suggesting that these are genes involved in normal liver functions that fail to function properly upon loss of *Per2*^–/^*^–^* or in early cancer stages (Figure 4C, cluster 3). We could not identify pathways upregulated in WT livers 1 week after cancer cell injection. In *Per2*^–/^*^–^* livers, however, we observed upregulation of genes involved in autophagy (*Ulk1, Plk3, Sesn2*), circadian regulation (*Ngfr, Klf10, Atf5*), and inhibition of cell proliferation (*Ngfr, Hes1, Tob1*) following cancer cell injection (Figure 4C and cluster 1 and Supplementary Table 2). This analysis suggests that in early stages of metastases formation, house-keeping genes are downregulated in WT livers, while only minor transcriptional upregulation is observed. Loss of *Per2*, however, rearranges the premetastatic liver niche, in a manner that activates tumor suppressing pathways in response to the presence of cancer cells, resulting in attenuated metastatic spread.

We further interrogated our RNA-sequencing data using CIBERSORT. This tool provides an estimation of the abundance of various cell types in a mixed cell population, based on expression of cell-type specific hallmark genes (Newman et al., 2019). We compiled several published single-cell datasets to create a reference dataset with which we examined the composition of our samples [Supplementary Table 3 and references (Heng and Painter, 2008; Dobie et al., 2019; Xiong et al., 2019)]. We found that the cellular composition is similar across samples (Supplementary Table 4), with one exception: cholangiocytes were significantly elevated in *Per2*^–/^*^–^* livers compared to WT livers (Supplementary Figure 2C and Supplementary Table 4). While this analysis did not highlight changes in cholangiocyte numbers between MC38 treated and non-treated livers, these findings suggest that *Per2* plays a role in cholangiocyte homeostasis. These findings are in line with previous reports showing that deletion of *Per2* exacerbates cholestatic liver injury and fibrosis (Chen et al., 2013). The majority of cells detected in all of our samples were hepatocytes (>95%, Supplementary Table 4), suggesting that transcriptional rewiring of these cells, rather than changes in cell composition, leads to the observed changes in gene expression.

### Loss of *Per2* in the Stroma Impairs Primary Tumor Growth

**FIGURE 5:**
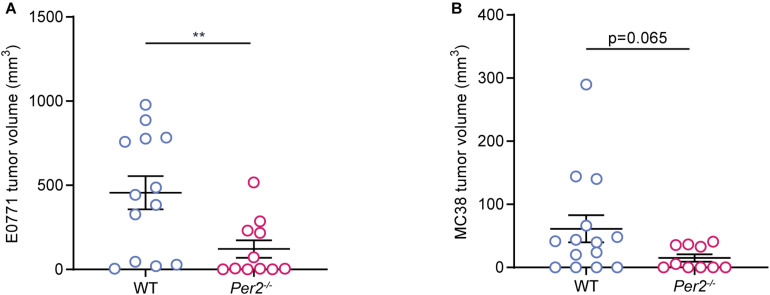
Stromal *Per2* controls primary tumor growth. **(A)** WT and *Per2*^–/^*^–^* mice were injected with E0771 cancer cells into the mammary fat pad. The mice were sacrificed at day 32, and tumor volume was calculated. *n* = 11–13 per genotype, combined from two independent experiments. **(B)** WT and *Per2*^–/^*^–^* mice were injected with MC38 cancer cells into the colon wall. Mice were sacrificed at day 14 and tumor volume was calculated. *n* = 10–14 per genotype combined from two independent experiments. *P*-value was calculated using unpaired student’s *t*-test with Welch’s correction. Error bars represent SEM, **p < 0.005.

Next, we asked whether stromal *Per2* is essential not only for metastatic colonization, but also for primary tumor formation. We used two orthotropic models: The E0771 triple negative breast cancer cell line injected to the mammary fat pad, and the MC38 colon cancer cell line (used for the liver metastasis model) injected into the colon submucosa by an endoscopy-guided procedure [as described in Zigmond et al. (2011)]. In both models, cancer cells were injected to *Per2*^–/–^ and WT mice, and tumor volume was assessed at the end point. *Per2*^–/–^ mice exhibited significantly smaller E0771 tumors compared to WT mice (Figure 5A), and a similar trend was seen for the MC38 colon model (Figure 5B). Taken together with our results from the liver metastasis model, these results suggest that stromal *Per*2 is required for tumor formation both in the primary site as well as in the metastatic site. These findings also imply that PER2 is required throughout the course of tumor progression and metastasis.

## Discussion

Disrupted circadian rhythms are generally thought to contribute to tumorigenesis and poor prognosis (Savvidis and Koutsilieris, 2012; Hadadi et al., 2019). However, much of the work performed on cancer and rhythmicity, to date, was done in a context in which both cancer cells and their microenvironment have disrupted clocks. Here we found that expression of the core clock gene *Per2* in the TME supports different stages of cancer progression. Employing different orthotopic tumor models, we showed that loss of stromal *Per2*, but not *Per1*, inhibits tumorigenesis and metastasis.

The circadian clock is a major regulator of metabolism and cell cycle and its disruption is well known to promote cancer. In cancer cells, both PER1 and PER2 were shown to act as tumor suppressors (Fu et al., 2002, 2016; Yang et al., 2009; Su et al., 2017; Zhu et al., 2019; Hou et al., 2020; Liu et al., 2020) and mutations in these genes were associated with human cancer (Chen et al., 2005; Kettner et al., 2014; Wu et al., 2019). However, whether these effects are clock-dependent is not clear. Moreover, despite the expansive knowledge of *Per1* and *Per2* roles in cancer cells, their roles in the TME were largely overlooked. Here we show that selective loss of the core clock genes *Per1* and *Per2* in the TME not only does not promote cancer but actually inhibits tumor growth and metastatic colonization. These findings suggest that the tumor-suppressive effects of PER1 and PER2 are most likely restricted to the cancer cells themselves, whereas in the TME PER1 and PER2 play different roles, that may be clock-independent.

The TME is comprised of different cell types. Which of these cell types contributes to the observed inhibitory effect of *Per2* loss on tumor growth and metastasis? Recent studies suggested an immune-regulatory role for *Per* genes in the TME, as mice with disrupted clocks have more protumorigenic immune microenvironments. Yet we find that T cell depletion does not compensate for the *Per1^–/–^Per2^–/–^*-driven inhibition of tumor growth, nor do we detect immune cell transcriptional signatures in early stages of metastases formation, suggesting that the adaptive immune microenvironment does not mediate this effect. Various other cell types play protumorigenic roles in the TME. We observed increased numbers of cholangiocytes in *Per2*^–/–^ mice, which may contribute to the anti-tumorigenic TME. Additionally, we detect massive transcriptional changes in hepatocytes of the pre-metastatic niche. Indeed, hepatocytes were recently reported to drive the formation of a pro-metastatic niche in the liver (Lee et al., 2019). CAFs were also proposed to play a role in clock regulation of cancer (Fuhr et al., 2019), and are generally known to have key roles in cancer progression. We observed massive infiltration of α-SMA-positive CAFs and collagen secretion in WT tumors which were not seen in *Per2*^–/–^ or *Per1^–/–^Per2^–/–^* mice. Whether this CAF infiltration is driven by *Per2* in fibroblasts, or perhaps by *Per2* in other cells of the TME, remains to be determined in future studies.

The liver is a circadian organ, which allows for the circadian regulation of different metabolic functions, such as the synthesis and metabolism of glucose, lipid, cholesterol, and bile acid (Bass and Takahashi, 2010; Ferrell and Chiang, 2015). These pathways are massively deregulated in *Per2*^–/–^ mice. Injection of cancer cells leads to further deregulation of these pathways, and induces other pathways (autophagy and inhibition of proliferation) which may contribute to the tumor-suppressive environment that prevents subsequent metastatic tumor growth. These processes may explain why, while PER2 serves as a tumor suppressor in cancer cells, it serves as a protumorigenic factor in the TME. In circadian biology, PER1, and PER2 function together to regulate clock functions. Our results show that *Per2*, but not *Per1*, plays an important role in the stromal regulation of tumor progression. These findings may imply a non-circadian role for *Per2*, differentiating it from *Per1*, and offering a new attractive therapeutic target in the TME.

## Materials and Methods

### Ethics Statement

All animal studies were conducted in accordance with the regulations formulated by the Institutional Animal Care and Use Committee (IACUC; protocols #02100220-2 and #02550418-3).

### Mice

*Per1^–/–^Per2^–/–^* (Zheng et al., 2001) were back crossed to C57BL/6 (Adamovich et al., 2019) and single *Per1^–/–^, Per2^–/–^*, were generated (kindly provided by Gad Asher, WIS). These mice and WT C57Bl/6 controls (ENVIGO RMS, Israel) were maintained under specific-pathogen-free conditions at the Weizmann Institute’s animal facility.

### Cell Culture

MC38 mouse colon cancer cells (kindly provided by Lea Eisenbach, WIS), LLC mouse lung carcinoma cells (kindly provided by Zvika Granot, HUJI), E0771 mouse breast cancer cells (kindly provided by Ronen Alon, WIS), and NIH3T3 mouse fibroblasts cell line (ECACC, #93061524) were cultured in Dulbecco’s modified Eagle’s medium (DMEM) (Biological Industries, 01-052-1A) supplemented with 10% fetal bovine serum (FBS) (Invitrogen), 1% pen-strep and 1% L-glutamine (Biological Industries). All cell lines were maintained at 37°C in 5% CO2 and regularly tested for mycoplasma by PCR assay (Biological Industries). For orthotropic and portal vein injections, the cells were suspended at 80–90% confluence by treatment with 0.25% trypsin, 0.02% EDTA and washed with 1x phosphate buffered saline (PBS).

### Generation of Per2::Luc Cells by Lentiviral Infection

pLenti6-B4B2 construct expressing *Per2*-*dLuc* (Liu et al., 2008) (kindly provided by Gad Asher, WIS) was inserted into MC38, LLC, NIH-3T3, and E0771 cells using lentiviral infection. After infection, the cells were selected by blasticidin-containing complete DMEM for 10 days.

### Real-Time Luminescence Monitoring

Cells were seeded into black, clear bottom 24-well plates (Provair) and synchronized with 100 nM Dexamethasone treatment for 20 min. Bioluminescence was measured by a LumiCycle luminometer (Actimetrics) every 12 min for at least 3 days in the presence of luciferin (Promega) in the cell culture medium. For siRNA experiments, 24 h after seeding, NIH3T3 Per2::luc cells were transfected with siRNA for the specified genes (SMART Pool siRNA, Dharmacon). After 24 h cells were synchronized by Dexamethasone and bioluminescence was measured as described above.

### Mathematical Analysis of Bioluminescence Data

To evaluate the rhythmicity of Per2::luc cells we created a CircadLib package for the MATLAB R2017b environment using a non-linear regression model. The model includes a constant level and one sinusoid to estimate the curve parameters – amplitude, phase and period. To remove effects of cell proliferation and signal decline the algorithm uses a de-trending function. To remove technical noise, the moving average method was used. This analysis yields two parameters: *R*^2^ – indicating how similar the curve is to a sinusoid, and π – indicating the period length.

### Quantitative RT–PCR Analysis

Total RNA was extracted from cells using Bio-Tri reagent (Bio-Lab). mRNA was reverse-transcribed using the High Capacity cDNA Reverse Transcription Kit (Applied Biosystems). Quantitative RT–PCR analysis was performed using Fast SYBR Green Master mix (Applied Biosystems) with the primers listed in Table 1.

**TABLE 1:** Primers used in this study.

	Forward	Reverse
HPRT	CATAACCTGGTTCATCATCGC	TCCTCCTCAGACCGCTTTT
TBP	CCCTATCACTCCTGCCACACCAGC	GTGCAATGGTCTTTAGGTCA
		AGTTTACAGCC
b2m	ACCGGCCTGTATGCTATCCAGAAA	GGTGAATTCAGTGTGA
		GCCAGGAT
Per1	ACCAGCCATTCCGCCTAAC	CGGGGAGCTTCATAACCAGA
Per2	GAAAGCTGTCACCACCATAGAA	AACTCGCACTTCCTTTTCAGG

### Liver Metastatic Colonization via Portal Vein Injection

Sixteen weeks old WT C57Bl/6, *Per1^–/–^, Per2^–/–^*, and *Per1^–/–^Per2^–/–^* mice, *n* = 9–16 per genotype, were injected with an analgesic agent (Buprenorphine, 0.1 mg/kg SC), and anesthetized using isoflurane 2.5%. To form liver experimental metastases, 20,000 MC38 cells were injected into the hepatic portal vein. To prevent bleeding, light pressure was applied to the injection site with a cotton applicator for 4 min. Then, the muscle and skin were sutured using 6/0 polypropylene monofilament sutures. At day 21 post-injection, mice were sacrificed, and body weights were measured. Livers were harvested and weighed, and macrometastases were quantified by visual inspection. To normalize liver weights, liver/body weight ratios were calculated. Then, livers were formalin-fixed and paraffin-embedded for histological analysis.

### Quantification of Liver Metastasis Analysis

Hematoxylin and Eosin (H&E) slides of liver tissues were scanned by a Pannoramic SCAN II scanner, 20×/0.8 objective (3DHISTECH, Budapest, Hungary). Quantification of liver metastases area was done by QuPath (version 0.2.0-m8) (Bankhead et al., 2017) with pixel classification using the simple threshold method, with prefilter Gaussian, smoothing sigma 4 and a threshold of 180.

### Total RNA Isolation From Livers of WT and *Per2*^–/–^ Mice

*Per2*^–/–^ or WT C57Bl/6 male mice were injected at the age of 16 weeks, under anesthesia, with 20,000 MC38 cells (*n* = 4 mice per genotype) and PBS (*n* = 2 control mice per genotype) into the hepatic portal vein as described above. At day 7 post-injection, mice were sacrificed, and body weights were measured. Livers were weighed and dissociated by Bead Ruptor Elite (OMNI International) with metal beads in Micro-tube (Sarstedt #72.694.006). RNA isolation was performed by RNeasy Fibrous Tissue Mini Kit (Qiagen #74704) according to manufacturer’s instructions.

### Library Preparation and RNA-Sequencing Analysis

Libraries were prepared using the SENSE mRNA-Seq Library Prep Kit V2 (Lexogen United States) according to the instructions of the manufacturer. Libraries were sequenced on an Illumina NovaSeq 6000 machine, at 10 M reads per liver sample, to provide sufficient reads to pass quality control (quality of reads and mapping quality percentage). Read counts were normalized and tested for differences using DEseq2 (Love et al., 2014). RNA sequencing results are detailed in Supplementary Table 1. Hierarchical clustering was performed using Euclidian distance on differentially expressed genes which were filtered with the following parameters: baseMean > 5, *p*adj < 0.05, and |log fold change| > 1. Analysis was performed by Partek Genomics Suite software. Pathway analysis was performed using Metascape (Zhou et al., 2019). Significant pathways were determined if *p* < 0.05 (for details see Supplementary Table 2). Liver populations analysis was performed using CIBERSORT (Newman et al., 2015) based on published single-cell RNA-sequencing data (Heng and Painter, 2008; Dobie et al., 2019; Xiong et al., 2019). The gene signatures of liver subpopulations used for this analysis and the results table are detailed in Supplementary Tables 3, 4, respectively. The RNA-seq data that support the findings of this study have been deposited in the Gene Expression Omnibus under accession number GSE156450.

### CD8+ CD4+ Depletion

WT C57Bl/6 and *Per1^–/–^Per2^–/–^* mice, *n* = 7–8 per genotype, were injected intraperitoneally with anti-mouse antibodies for CD8 (Clone 53–6.7) and CD4 (Clone GK1.5) or IgG2a control (Clone 2A3; 300 μg per injection per mouse for all antibodies; Bio X Cell), 2 days prior to the injection of MC38 cells to the portal vein as described above, and every 5 days to maintain full depletion until the endpoint. 50 μl blood were drawn to validate depletion of CD8+ and CD4+ cells by flow cytometry. At day 21, mice were sacrificed, body weights were measured, livers were fixed with 4% PFA, and macrometastases were quantified by visual inspection.

### Orthotropic Injection to the Colon

*Per2*^–/–^ or WT C57Bl/6 male mice, *n* = 10–14 per genotype, were injected at the age of 16 weeks, under anesthesia, with 200,000 MC38 cells suspended in 20 μl PBS into the colon wall (submucosal). The injection was performed under endoscopic guidance. Body weights were measured using an analytical balance, the appearance of tumors was monitored by colonoscopy at days 10 and 14. At day 14, mice were sacrificed, colons were harvested, and tumor volumes were measured using a digital caliper and calculated using the formula: *V* = (W^2^ × L)/2.

### Orthotropic Injection to the Mammary Fat Pad

*Per2*^–/^*^–^* or WT C57Bl/6 female mice, *n* = 11–13 per genotype, were injected at the age of 8–11 weeks, under anesthesia, with 200,000 E0771 cells into the upper left mammary fat pad. Body weights were measured, and tumor volumes were measured using a digital caliper and calculated using the formula: *V* = (W^2^ × L)/2. Mice were sacrificed at day 32.

### Immunohistochemistry of Mouse Tissues

Livers were fixed in 4% Paraformaldehyde (PFA), processed and embedded in paraffin blocks, cut into 4–5 μm sections and immunostained as follows: Formalin-fixed, paraffin-embedded (FFPE) sections were deparaffinized, treated with 0.3% H_2_O_2_ and antigen retrieval was performed by microwave with citrate acid buffer (pH 6.0). Slides were blocked with 10% normal horse serum, and anti-SMA antibodies were used (Sigma #F3777). Visualization was achieved with 3, 30-diaminobenzidine (DAB) as a chromogen (#SK4100, Vector Labs Kit, CA, United States)/M.O.M Kit (Vector Labs). Counterstaining was performed with Mayer’s Hematoxylin (MHS-16, Sigma-Aldrich, Rehovot, Israel). Images were taken with a Nikon Eclipse Ci microscope.

### Statistical Analysis

Statistical analysis and visualization were performed using R (Version 3.6.0, R Foundation for Statistical Computing, Vienna, Austria) and Prism 8.2.0. (GraphPad, United States). Statistical tests were performed as described in each Figure Legend.

## Data Availability Statement

The original contributions presented in the study are publicly available. This data can be found here: https://www.ncbi.nlm.nih.gov/geo/query/acc.cgi?acc=GSE156450.

## Ethics Statement

The animal study was reviewed and approved by the Weizmann Institute of Science Institutional Animal Care and Use Committee.

## Author Contributions

LS, SM, and CL designed and performed the experiments and analyses and wrote the manuscript. HL performed the experiments. AN wrote code for mathematical analysis of bioluminescence data and assisted with writing the manuscript. RS-S designed and supervised the study and wrote the manuscript. All authors contributed to the article and approved the submitted version.

## Conflict of Interest

The authors declare that the research was conducted in the absence of any commercial or financial relationships that could be construed as a potential conflict of interest.
