# Supplementary figures and images for: Stromal Expression of the Core Clock Gene Period 2 Is Essential for Tumor Initiation and Metastatic Colonization
Source: Front Cell Dev Biol. 2020 Oct 2;8:587697. doi: 10.3389/fcell.2020.587697 (PMC7573548; doi:10.3389/fcell.2020.587697)

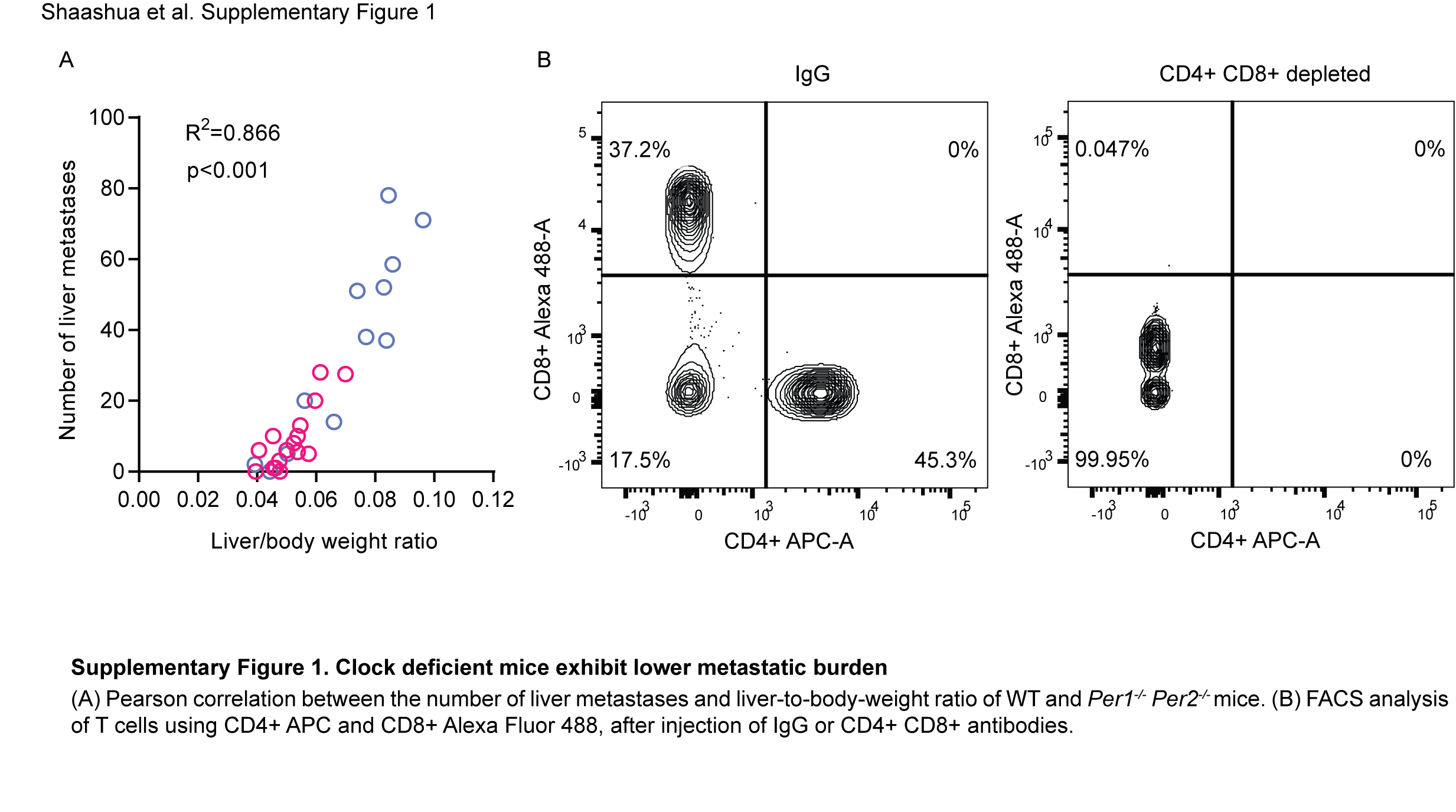

Supplement: Supplementary file 1 [file Image_1.TIF]

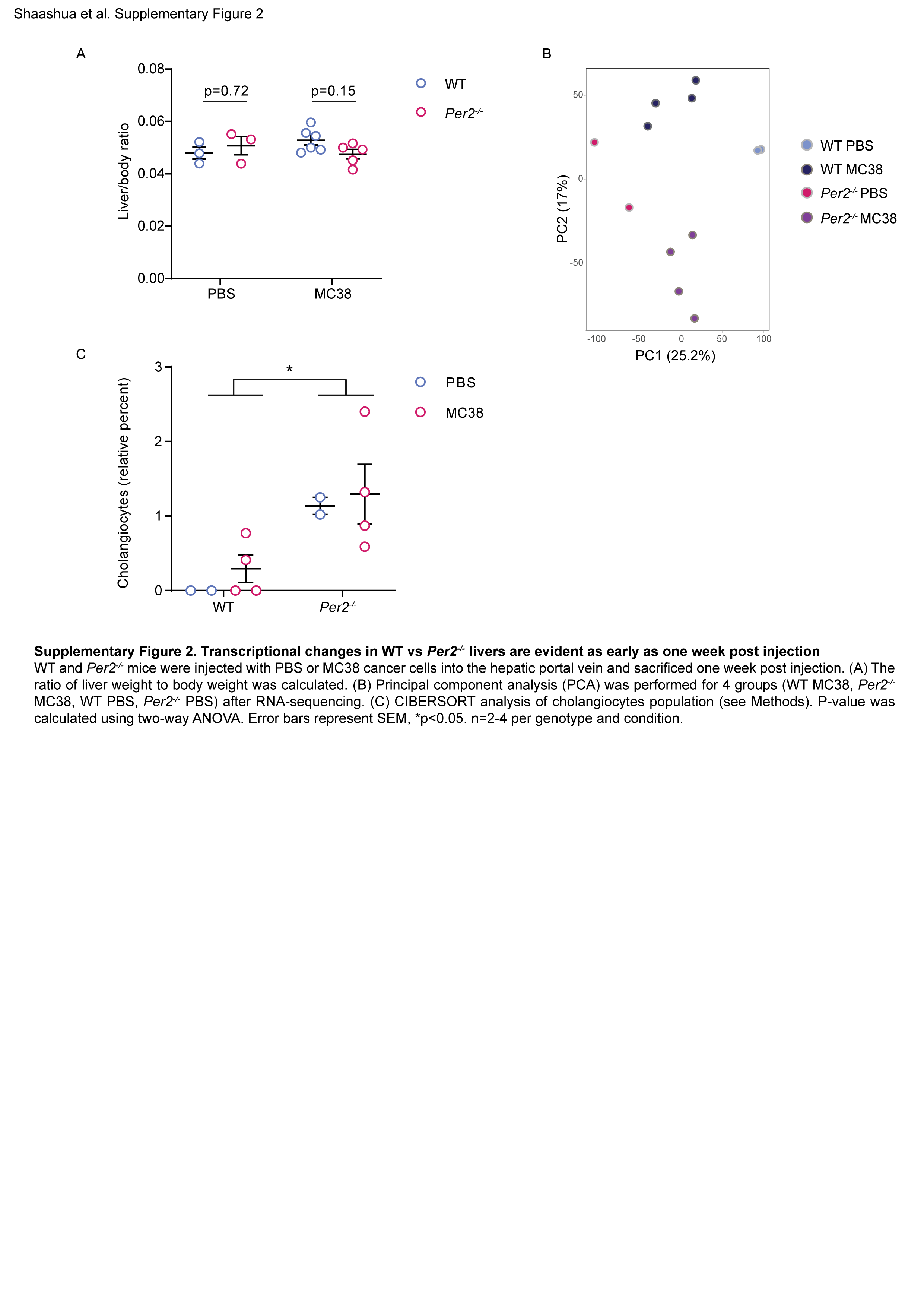

Supplement: Supplementary file 2 [file Image_2.TIF]
